# Supplementary material for: AR/ER Ratio Correlates with Expression of Proliferation Markers and with Distinct Subset of Breast Tumors
Source: Cells. 2020 Apr 24;9(4):1064. doi: 10.3390/cells9041064 (PMC7226480; doi:10.3390/cells9041064)
Supplement: Supplementary file 1 [file cells-09-01064-s001.pdf]

SUPPLEMMENTARY MATERIAL

Supplementary Figure 1. Correlation between protein and mRNA expression for AR and ER in ER+/AR+ BC cases. IHC: Immunohistochemistry. FC: Fold Change. qPCR: Real time quantitative PCR. *Spearman’s correlation test.

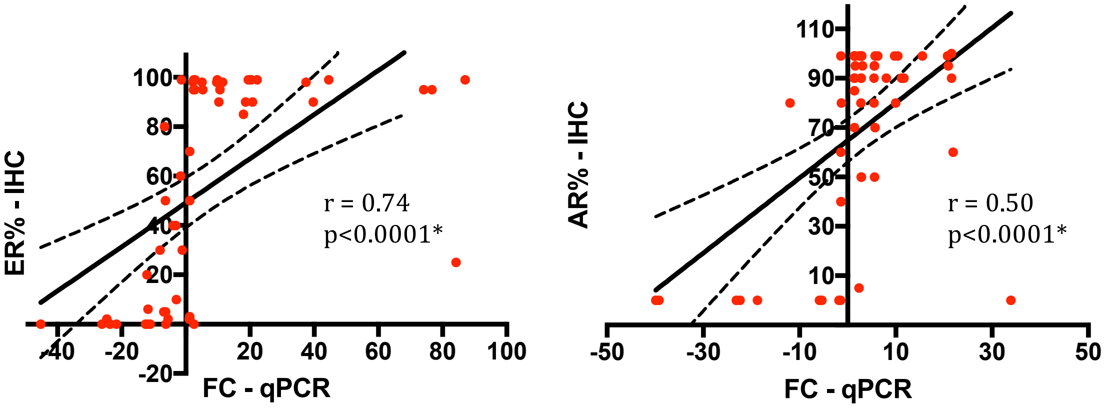

Supplementary Figure 2. Protein expression of the proliferation marker Ki-67 in BC cases studied. Significant differences in expression of Ki-67 were not observed in BC cases grouped by AR/ER ratio, neither when cases were divided by ER% positivity levels. ER-: Estrogen Receptor – Negative = <1%. *Tukey`s multiple comparison test. ns: Non significant.

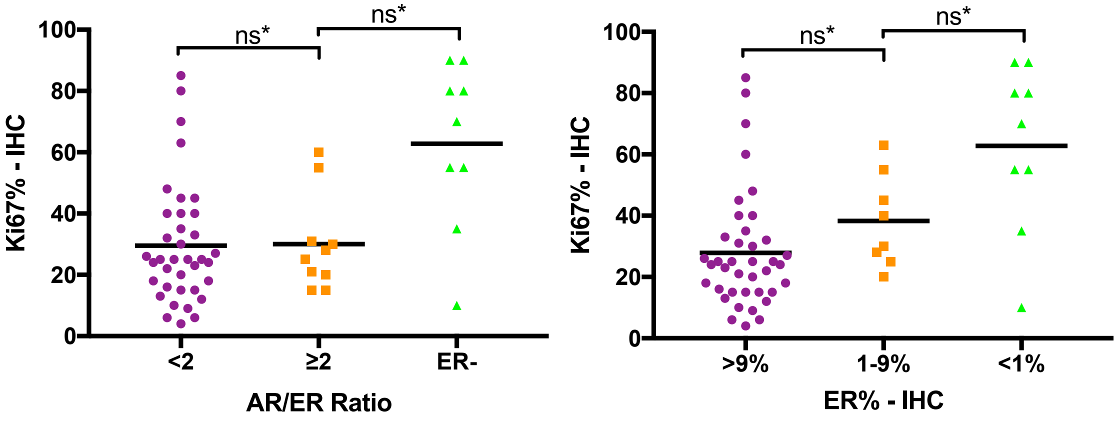

Supplementary Figure 3. Correlations between qPCR-MKI67 levels and Ki67-IHC score in all cases (A), AR/ER<2 group (B) and AR/ER≥2 group (C). IHC: Immunohistochemistry. FC: Fold Change. qPCR: Real time quantitative PCR. *Spearman's correlation test.

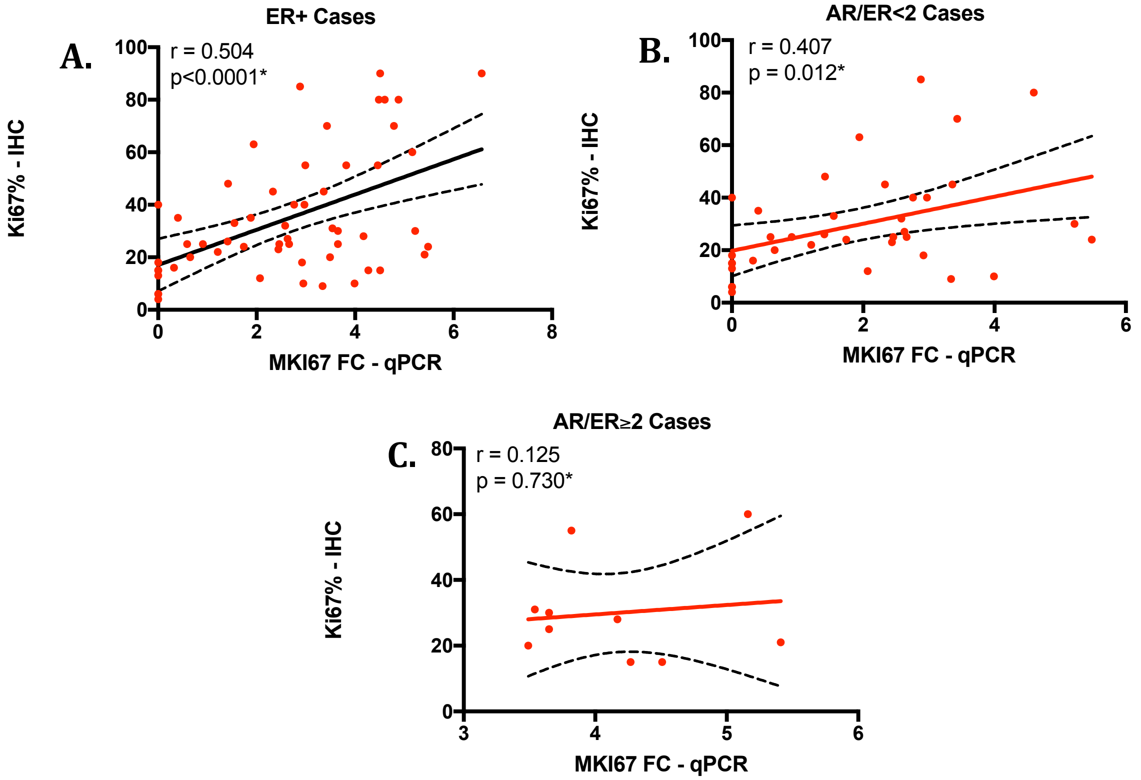

Supplementary Figure 4. Correlations between CPS score and Ki67-IHC score in all cases (A), AR/ER<2 group (B) and AR/ER≥2 group (C). CPS: Cell Proliferation Signature. IHC: Immunohistochemistry. FC: Fold Change. qPCR: Real time quantitative PCR. *Spearman's correlation test.

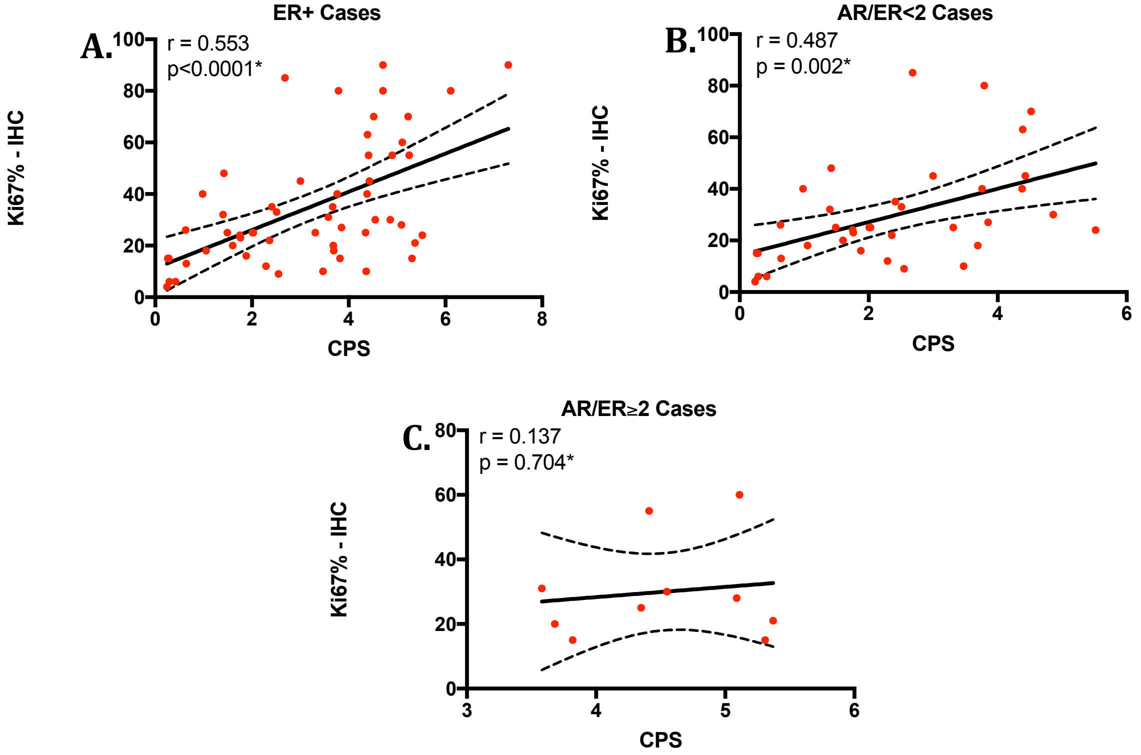

Supplementary Table 1. Clinical and histopathological characteristics of 979 breast cancer patients from GDC database (External validation cohort). CPS (Cell Proliferation Signature), IDC-NST (Invasive Ductal Carcinoma – Non Special Type), ILC (Invasive Lobular Carcinoma). ER (Estrogen Receptor). AR (Androgen Receptor). PgR (Progesterone Receptor). **CPS:** *AURKA, BIRC5, CCNB1, MKI67* and *UBE2C*. **CPS2:** *AURKb, BUB1B, BUB1, CDK1* and *CHEK*

Characteristics		N (%)	CPS	*p*	CPS2	*p*	ER +		*p*	ER -	*p*
							AR/ER < 2	AR/ER ≥ 2			
Age	<50	291 (29.7)	0.487	< 0.0001	0.246	< 0.0001	178 (25.5)	24 (55.8)	< 0.0001	89 (37.6)	< 0.0001
	>50	688 (70.3)	0.214		0.011		521 (74.5)	19 (44.2)		148 (62.4)	
Tumor Size	<20 mm	251 (25.6)	-0.003	< 0.0001	-0.132	< 0.0001	189 (27)	11 (25.6)	0.843	51 (21.5)	0.243
	≥20 mm	728 (74.4)	0.398		0.154		510 (73)	32 (74.4)		186 (78.5)	
Lymph node involvement	pN0	462 (49.7)	0.339	0.173	0.117	0.159	315 (45.9)	16 (38.1)	0.403	131 (55.7)	0.05
	pN1-3	435 (44.4)	0.282		0.076		321 (46.7)	24 (57.1)		90 (38.3)	
	pN>3	68 (6.9)	0.116		-0.087		51 (7.4)	2 (4.8)		14 (6.0)	
Histological Grade	1	173 (17.7)	0.001	< 0.0001	-0.136	< 0.0001	128 (18.7)	8 (19)	0.92	31 (13.4)	0.012
	2	557 (56.9)	0.398		0.172		375 (54.7)	29 (69.0)		153 (65.9)	
	3	235 (25.4)	0.282		0.025		183 (26.6)	5 (11.9)		48 (20.7)	
Histotype	IDC-NST	727 (74.3)	0.457	< 0.0001	0.225	< 0.0001	483 (69.1)	35 (81.4)	0.893	209 (88.2)	< 0.0001
	ILC	187 (19.1)	-0.299		-0.442		170 (24.3)	7 (16.3)		10 (4.2)	
	Mixed type	15 (1.5)	-0.144		-0.349		15 (2.1)	0 (0.0)		0 (0.0)	
	others	50 (5.1)	0.285		0.115		331 (4.5)	1 (2.3)		18 (7.6)	
Vascular invasion	No	839 (97.4)	0.344	0.318	0.122	0.151	562 (97.2)	37 (97.4)	0.96	210 (98.1)	0.774
	Yes	22 (2.6)	0.553		0.149		16 (2.8)	1 (2.6)		4 (1.9)	
PgR	0	319 (32.7)	0.987	< 0.0001	0.715	< 0.0001	89 (12.7)	14 (32.6)	0.001	216 (91.1)	> 0.0001
	>1%	656 (67.3)	-0.043		-0.227		607 (86.8)	29 (67.4)		20 (8.4)	
HER2	Negative	712 (80.2)	0.263	0.031	0.06	0.145	526 (83.2)	20 (48.8)	< 0.0001	166 (77.2)	< 0.0001
	Positive	176 (19.8)	0.409		0.144		106 (16.8)	21 (51.2)		49 (22.8)	
AR/ER	≥2	43 (5.8)	0.232	< 0.0001	0.025	< 0.0001					
	< 2	699 (94.2)	-0.033		-0.230						
ER	0	237 (24.2)	1.312	< 0.0001	1039,00	< 0.0001					
	>1%	742 (75.8)	-0.006		-0.204						
